# Non-Specific Strength Changes Between High- and Low-Load Isotonic Resistance Training: A Systematic Review and Meta-Analysis

**DOI:** 10.1007/s40279-025-02370-8

**Published:** 2025-12-12

**Authors:** William B. Hammert, Ryo Kataoka, Yujiro Yamada, Robert W. Sallberg, Anna Kang, Samuel L. Buckner, Jeremy P. Loenneke

**Affiliations:** 1 Kevser Ermin Applied Physiology Laboratory, Department of Health, Exercise Science, and Recreation Management, The University of Mississippi, P.O. Box 1848, University, MS 38677 USA; 2 Exercise Science Program, USF Muscle Lab, University of South Florida, Tampa, FL USA

## Abstract

**Background:**

In comparisons between high- and low-load isotonic resistance training, it has become common to include non-specific strength tests (e.g., isometric and isokinetic strength tests), presumably in attempt to minimize the influence of training specificity and better understand the efficacy of low-load training for developing maximal strength. Many have suggested that high- and low-load isotonic resistance training are similarly effective for increasing non-specific strength, provided exercise is performed to task failure. However, little work has been completed to examine the accuracy of such statements.

**Objective:**

We aimed to quantitatively identify whether high-load isotonic resistance training results in differential changes in non-specific strength compared to low-load isotonic training.

**Methods:**

A systematic search of the literature was conducted using PubMed, Scopus, and Embase from inception to 14 June, 2025. To be included in the present review, a study needed to: (a) be performed in healthy adult humans ≥ 18 years of age; (b) include isotonic high- and low-load isotonic resistance training protocols that were prescribed to task failure; (c) have measured non-specific strength at both pre- and post-intervention via an isometric or isokinetic maximum strength task; (d) matched the number of strength tests between the high- and low-load training groups; (e) be published in a peer-reviewed journal; and (f) published in the English language. A random-effect meta-analysis using robust estimation variation was then implemented on the changes (i.e., pre- to post-intervention) in non-specific strength between high- and low-load isotonic resistance training.

**Results:**

The literature search yielded 7885 unique articles, of which ten studies were selected for inclusion in the present analysis. Using effect size values calculated from the change score standard deviations resulted in 44 ES from ten studies (245 total participants; high load, *n* = 114; low load, *n* = 131). The overall effect size (Cohen’s *d*) was 0.322 with a standard error of 0.17, a 95% confidence interval of − 0.08 to 0.72 (*p* = 0.104), and 95% prediction intervals that ranged from − 0.45 to 1.1. A supplemental analysis using pre-standard deviations resulted in similar conclusions.

**Conclusions:**

The results of the current analysis were inconclusive as to whether high- and low-load isotonic training induced differential changes in non-specific strength. The overall effect size appeared to be biased towards favoring high-load isotonic training; however, the confidence intervals were wide and crossed zero.

**Supplementary Information:**

The online version contains supplementary material available at 10.1007/s40279-025-02370-8.

## Key Points

**Table:** 

Previous research has consistently shown that higher load resistance training is more effective than low-load training for increasing task-specific strength, which can likely be explained by adaptations underlying the principle of training specificity.
It becomes much more difficult to detect and predict whether changes in strength will favor high- or low-load isotonic training when assessed using less familiar, “non-specific” strength tasks (i.e., isometric and isokinetic strength tests).
While the overall effect between high- and low-load isotonic training appeared to be biased towards favoring high-load isotonic training, the results of the current analysis were inconclusive. Additional research implementing a time-matched non-exercise control group and a direct comparison between low- and high-low isotonic resistance training without retesting maximal strength is warranted.

## Introduction

Muscular strength describes the ability to produce maximum force against an external resistance [1] and can be assessed using different performance tasks (e.g., isometric [2, 3], isokinetic [4, 5], one-repetition maximum [1RM] [3, 6] strength tests). Irrespective of the assessment, the ability to increase muscular strength through resistance training appears to be influenced by repeated motor patterns and/or skill acquisition associated with practicing a specific exercise (i.e., training specificity) [2, 7–11]. For example, a recent meta-analysis [8] found that isotonic resistance training increased both “task-specific” strength (i.e., isotonic strength tested in the same exercise as the training intervention) and “non-specific” strength (i.e., isokinetic or isometric strength tested on a device that was not used during the training intervention) compared with a non-exercise control group. Yet, the magnitude of change in task-specific strength was much larger than that of non-specific strength changes [8], which is consistent with the principle of training specificity. As it pertains to maximum isometric handgrip strength, Wong et al. [11] reported that exercising at 100% intensity (i.e., 4 sets of maximal contractions) resulted in greater strength increases compared with 30% training intensity [11]. Likewise, in studies that have examined strength adaptations between isotonic high- and low-load resistance training, it has been comprehensively demonstrated that higher load training produces greater increases in isotonic 1RM strength in exercises that were performed during the intervention [7, 10, 12, 13].

Despite a large body of evidence indicating that high-load resistance training is more effective than low-load training for increasing task-specific strength [7, 11–13], it becomes much more difficult to detect changes [8] and predict whether adaptations will favor high- or low-load training when strength changes are assessed using less familiar tasks (i.e., non-specific strength tests) [14]. Whereas some researchers have observed that high-load isotonic training led to greater improvements in non-specific strength (i.e., isometric and isokinetic strength) than low-load isotonic training [15–17], others have documented similar changes following periods of high- and low-load isotonic training [4, 18, 19]. Many will interpret a lack of difference in non-specific strength changes to mean that high- and low-load isotonic resistance training to task failure are similarly effective for increasing strength [18, 20–22]. Indeed, an evidence-based review [21] on the topic of training for strength adaptations concluded that any form of resistance training is effective for increasing non-specific strength, and that heavier loads are not superior than lower loads when muscular strength is evaluated using non-specific strength tests. Likewise, in a paper discussing the implications of lower load training for health and performance, Weakley et al. [20] proposed that low-load training should be considered a viable alternative to higher load training, in part, based on data from Mitchell et al. [18] who reported similar changes in non-specific strength between high- and low-load isotonic training.

Previous meta-analyses [13, 23, 24] examining the effects of high- and low-load isotonic resistance training on strength adaptations have primarily focused on changes in 1RM strength. Schoenfeld et al. [12] also included a comparison between high- and low-load isotonic training on changes in isometric strength, but were unable to quantify the effects on changes in isokinetic strength. Moreover, the Schoenfeld et al. meta-analysis [12] incorporated data from within-subject experimental designs, which is likely problematic, as it has been shown that high-load training in one limb can influence the magnitude of strength gain in the opposite limb undergoing lower load training [25]. Motivated by these shortcomings, as well as the conflicting results between high- and low-load isotonic resistance training on changes in non-specific strength [4, 15–19, 22], the current project aimed to quantitatively identify whether high-load isotonic resistance training results in differential changes in non-specific strength compared to low-load isotonic training. A meta-analysis was implemented on the changes (i.e., pre- to post-intervention) in isometric and isokinetic strength following high- and low-load isotonic resistance training.

## Methods

### Search Strategy

**Fig. 1:**
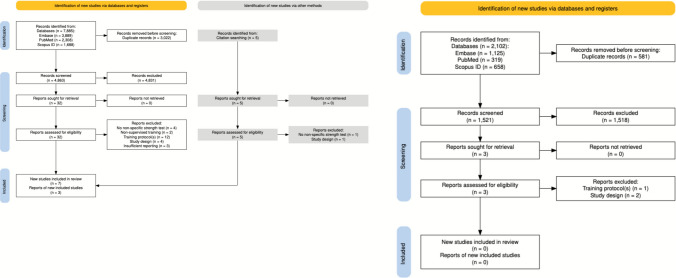
Preferred Reporting Items for Systematic reviews and Meta-Analyses (PRISMA) flowchart of the study selection process, per Haddaway et al. [48]. The left panel represents the original search, and the right panel represents the updated search

A systematic search of the literature was conducted using PubMed, Scopus, and Embase from inception to 1 March, 2024 (Fig. 1). Our search strategy was molded from previous reviews and meta-analyses [7, 12, 23] published on the topic of high- versus low-load resistance training. An initial check of the search terms “high and low load” and “resistance training” was employed and where appropriate, additional keywords added, and modifications made, in attempt to obtain an expansive and complete literature search. Articles were identified through Embase (*n* = 3889) with the following search terms from all fields: (((high OR heavy) AND load OR ((high OR heavy) AND intensity)) AND (low OR light) AND load OR ((low OR light) AND intensity)) AND (resistance OR strength OR weight) AND training NOT (animal OR disease), through PubMed (*n* = 2308) with the following search terms from all fields: (((high load OR high-load OR high intensity OR high-intensity OR heavy load) AND (low load OR low-load OR low intensity OR low-intensity OR light load)) AND (resistance training OR resistance exercise OR strength training OR weight training)) NOT ((animal OR disease)), and through Scopus (*n* = 1688) with the following search terms within the article title, abstract, and keywords: ‘high AND load OR high-load’ OR ‘high AND intensity OR high-intensity’ OR ‘heavy AND weight’ AND ‘low AND load OR low-load’ OR ‘low AND intensity OR low-intensity’ OR ‘light AND weight’ AND ‘resistance AND training’ OR ‘resistance AND exercise’ OR ‘strength AND training’ OR ‘weight AND training’ AND NOT ‘animal OR disease’. After the initial literature search was completed, the articles were pooled into Rayyan (Qatar Computing Research Institute Data Analytics), an online tool allowing reviewers to include and exclude articles and provide a rationale behind each decision [26], and manually screened for eligibility. A supplemental examination of the references of identified articles (e.g., meta-analyses, systematic reviews) on the topic of high- versus low-load resistance training was subsequently completed, and any overlooked studies were added in manually. Because of the lengthy peer-review process, it was suggested that we reopen the literature search to capture any newly published studies and enhance the comprehensiveness of our analysis. In response, we conducted an updated search using the same search terms and inclusion criteria as the original review. The updated search covered studies published between 1 March, 2024 and 14 June, 2025, and yielded a total of 2102 additional articles across the three databases: Embase (*n* = 1125), PubMed (*n* = 319), and Scopus (*n* = 658).

### Eligibility Criteria

For a study to be included in the present review, it needed to meet the following criteria: (a) performed in adult humans ≥ 18 years of age with no known medical conditions or musculoskeletal injuries; (b) included an isotonic high- and low-load resistance training protocol(s) (i.e., repeated bouts of isotonic dynamic resistance exercise lasting at least 4 weeks and a minimum of ten training sessions) that were prescribed to task failure; (c) measured non-specific strength at both pre- and post-intervention via an isometric or isokinetic maximum strength task; (d) matched the number of strength tests between the high- and low-load training groups; (e) published in a peer-reviewed journal; and (f) published in the English language.

We elected to only include studies that employed training to task failure, to ensure that the stimulus was maximized and comparable across loading schemes. Such an approach was chosen to address our research question within the context of the available literature. High-load resistance training was operationally as exercise loads > 60% 1RM strength, whereas low-load resistance training was defined as ≤ 40% 1RM strength. Our operational definition of high-load resistance training was selected to apply terminology similar to that of Schoenfeld et al. [12] and to align with the American College of Sports Medicine’s minimum threshold (≥ 60% 1RM) for eliciting strength adaptations. When loading was reported as the number of repetitions performed (e.g., 8–12RM), rather than as percentage of 1RM, all repetitions up to a 15RM were classified as high-load training, whereas repetitions exceeding a 15RM were classified as low-load training, consistent with Schoenfeld et al. [12].

A study was excluded from the present review if it: (a) employed a within-subject study design (i.e., unilateral exercise model), whereby each side of the body (e.g., right arm) is subjected to one training protocol, and the contralateral side of the body (e.g., left arm) undergoes a separate exercise intervention; (b) only used alternative forms of dynamic isotonic resistance training (e.g., blood flow restriction training, elastic band training, electrostimulation training, bodyweight exercises); (c) prescribed, or allowed, participants to concurrently perform any other type of exercise (e.g., aerobic exercise training, sport); (d) measured non-specific strength on the same device that participants trained on, or in a muscle (group) that was not involved in the training (e.g., trained leg press and tested handgrip strength); (e) prescribed different exercises between training protocols (e.g., one group performed push-ups and the other performed the bench press); (f) did not prescribe both high- and low-load training protocols to task failure; (g) assigned participants to a weight loss/dieting intervention (e.g., hypo-energetic caloric intakes); (h) afforded participants supplements (e.g., creatine monohydrate, beta-alanine) with the exception being protein, which needed to be matched between groups; and (i) did not report sufficient data to calculate an effect size (ES) for our outcome variables of interest. Table S1 in the Electronic Supplementary Material (ESM) shows the excluded studies as well as the specific reason they were excluded.

### Statistical Analyses

The data were analyzed by WBH and JPL. With the exception of one study [17], which required data to be extracted using a graph digitizer (https://apps.automeris.io/wpd/), all data were obtained from the results section of the study and/or personal communication with the study’s lead author by WBH and RK. We specifically pooled changes in isometric and isokinetic strength into a single analysis to represent a “non-specific” strength outcome. Such an approach has been used previously [8] and aligns with the commonly stated claim that when the assessment is not specific to the training exercise, strength development is similar between low- and high-load resistance training [18, 20–22]. Effect size values were calculated for each study using the mean difference and the standard deviation (SD) of the difference (commonly known as Cohen’s dz), as recommended by Dankel and Loenneke [27]. If the SD was not reported, but an exact *p*-value was, then the *t*-value was calculated using the inverse of the cumulative distribution function. The *t*-value was then used to calculate the change score SD. We chose to normalize the mean difference to the SD of the difference, namely because we were interested in capturing the magnitude of the variability within the intervention itself. As it may be of interest to some, we also reran the analysis using the mean difference normalized to the pre-SDs (provided in Fig. S1 of the ESM). In terms of the former, if the variability of the change (i.e., the SD of the change) was not provided, it was estimated using the following formula:

$${\mathrm{SD}}_{\text{of change}}=\sqrt{[({{\mathrm{SD}}_{\mathrm{pre}-\mathrm{test}})}^{2}+({{\mathrm{SD}}_{\mathrm{post}-\mathrm{test}})}^{2}-\left(2r\times {\mathrm{SD}}_{\mathrm{pre}-\mathrm{test}}\times {\mathrm{SD}}_{\mathrm{post}-\mathrm{test}}\right)] }.$$

An *r* value of 0.9 was the pre-post correlation for all estimations, as previous studies have reported large pre- to post-test correlations (e.g., 0.85–0.99) on upper and lower body strength tests [5, 28–30]. The standardized ES and standard error (SE) of the standardized ES were computed as follows [31]:

$$\text{Standardized ES}=\frac{{\mathrm{Change}}_{\text{High Load}}- {\mathrm{Change}}_{\text{Low Load}}}{\sqrt{ \frac{{(N}_{1}-1){v}_{1}+ {(N}_{2}-1){v}_{2} }{{N}_{1} + {N}_{2} - 2}}},$$

$$\text{ SE}=\sqrt{\frac{{N}_{1} + {N}_{2} }{{N}_{1} * {N}_{2}}+ \frac{{\mathrm{ES}}^{2} }{{2(N}_{1}+ {N}_{2})}}.$$

All statistics were computed using the robumeta package (version 2.0) within R Studio (version 1.4.1717). We implemented the robumeta package in order to account for dependency between ESs [32]. All studies were weighted using the inverse variance weight, and ESs are reported in standardized units (Cohen’s *d*). We ran a correlated effects model with small-sample corrections. The default correlation was 0.8. However, we also ran a sensitivity analysis to determine the effect of rho on tau squared. Prediction intervals were also included to provide information on where the ES of a new study would fall if the study was selected at random from the same population of the studies already included in the meta-analysis [33]. Forest plots were included to provide point estimates of the individual ESs in graphical form as boxes with 95% confidence intervals surrounding each block. Values above and below 0 favored high- and low-load isotonic training, respectively. The overall effect is included at the bottom of the plot as a diamond with a width equivalent to the confidence interval for the estimated effect (via the forest.robu function in the robumeta package).

### Assessment of Risk of Bias

The risk of bias in individual studies was evaluated according to the second version of the Cochrane risk-of-bias tool for randomized trials (RoB 2) [34], which focuses on different aspects of trial design, conduct, and reporting. Each assessment using the RoB 2 tool is focused on the outcome level. The six-item instrument used to evaluate each included study is as follows: (1) randomization process; (2) deviation from intended interventions; (3) missing outcome data; (4) measurement of the outcome; (5) selection of the reported result; and (6) overall analysis. Overall risk of bias was expressed as “low risk of bias” (i.e., if all domains were classified as low risk), as “some concerns” (i.e., if some concern was raised in at least one domain but not classified as high risk in any other), or as “high risk of bias” (i.e., if at least one domain was classified as high risk, or there were multiple domains with some concerns) [34]. Two independent researchers (WBH and RK) evaluated the studies and any disagreement was then reconciled.

## Results

The literature search yielded a total of 7885 articles. After deduplication, the titles and abstracts of 4863 articles were screened, upon which the 32 articles that satisfied the title and abstract screening process were printed, independently read, and reviewed. Seven of the 32 studies met the inclusion criteria, and an additional three studies were added from the supplemental examination of previous literature (i.e., citation searching); hence, a total of ten studies were selected for inclusion in the analysis (Fig. 1). The updated literature search yielded a total of 2102 additional articles. After removing 581 duplicates, 1521 unique articles remained for title and abstract screening. Of these, three studies were identified as potentially eligible and were retrieved for a full-text review. However, none met the predefined inclusion criteria. The characteristics (i.e., population, training intervention) of the ten studies are summarized in Table 1.

**Table 1:** Characteristics of the studies included in the present analysis

Study	Population	Training intervention	Training protocols	Non-specific strength test(s)
Bello et al. 2024 [38]	Trained male individuals 20.4 ± 2.7 years	9 weeks, 3 ×/week: Total body	A. 3 × Failure, 85% 1RM; 2 min (*n* = 8) B. 3 × Failure, 30% 1RM; 2 min (*n* = 9)	Unilateral isometric right, left KE, 90° Unilateral isometric right, left KF, 90° Unilateral isokinetic right, left KE, 60°/s Unilateral isokinetic right, left KE, 120°/s Unilateral isokinetic right, left KF, 60°/s Unilateral isokinetic right, left KF, 120°/s
Colomer-Poveda et al. 2020 [16]	Untrained male individuals 21.0 ± 1.3 years	4 weeks, 4 ×/week: Unilateral KE	A. 3 × Failure, 75% 1RM; 2 min (*n* = 11) B. 3 × Failure, 25% 1RM; 2 min (*n* = 11)	Unilateral isometric KE, 90
De Sousa et al. 2023 [46]	Untrained female individuals 58.5 ± 8.0 years	12 weeks, 3 ×/week: KE, LC, LP, SCR	A. 1–3 × Failure, ~ 80% 1RM; 90 s (*n* = 15) B. 1–3 × Failure, ~ 30% 1RM; 90 s (*n* = 17)	Isometric KE, 70°
Hisaeda et al. 1996 [39]	Untrained female individuals 20.1 ± 1.6 years	8 weeks, 3 ×/week: KE	A. 8–9 × 4–5RM; --*-* (*n* = 6) B. 5–6 × 15–20RM; 90 s (*n* = 5)	Isometric KE, 80° Isokinetic KE, 60, 180, 300°/s
Jenkins et al. 2015 [40]	Untrained male individuals 21.7 ± 2.4 years	4 weeks, 2–3 ×/week: dumbbell BC	A. 3 × Failure, 80% 1RM; 2 min (n = 7) B. 3 × Failure, 30% 1RM; 2 min (*n* = 8)	Isometric EF, 90°
Jenkins et al. 2017 [17]	Untrained male individuals 23.1 ± 4.7 years	6 weeks, 3 × /week: LE	A. 3 × Failure, 80% 1RM; 2 min (*n* = 13) B. 3 × Failure, 30% 1RM; 2 min (*n* = 13)	Isometric LE, 90°
Lim et al. 2019 [22]	Untrained male individuals 23.5 ± 1.61 years	10 weeks, 3 ×/week: LP, LE, LC	A. 3 × Failure, 80% 1RM; --*-* (*n* = 7) B. 3 × Failure, 30% 1RM; (*n* = 7)	Unilateral isokinetic KE, 60, 240°/s
Van Roie et al. 2013 [37]	Untrained individuals 65.6 ± 4.8 years	12 weeks, 3 ×/week: LP, LE	A. 2 × 10–15RM, 80% 1RM; 1 min (*n* = 18) B. 1 × 80–100RM (*n* = 19) C. 1 × 60 + 10–20RM, 20–40% 1RM (*n* = 19)	Unilateral isometric KE, 90, 120, 150° Unilateral isokinetic KE, 60, 180, 240°/s
Van Roie et al. 2013 [41]	Untrained individuals 21.8 ± 1.9 years	9 weeks, 3 ×/week: bilateral LE	A. 1 × 10–12RM, 80% 1RM (*n* = 12) B. 1 × 60 + 10–12RM, 20–40% 1RM (*n* = 12)	Unilateral isometric KE, 90° Unilateral isokinetic KE, 60°
Weiss et al. 1999 [45]	Untrained male individuals 21.1 ± 2.1 years	7 weeks, 3 ×/week: barbell squat	A. 4 × 3–5RM; --*-* (*n* = 7) B. 4 × 13–15RM; --*-* (*n* = 10) C. 4 × 23–25RM; --*-* (*n* = 11)	Unilateral isometric KE, 60, 300° Unilateral isokinetic KF, 60, 300°/s

Population: *Trained,* resistance-trained individuals, *Untrained*, non-resistance-trained individuals. Training intervention: study duration (weeks), number of training sessions per week: exercise(s) prescribed. Training protocols: sets × repetitions, load; inter-set rest interval (number of participants)

*1RM* one-repetition maximum, *A* Training Protocol “A”, *B* Training Protocol “B”, *BC* biceps curls, *C* Training Protocol “C”, *EF* elbow flexion, *KE* knee extension, *KF* knee flexion, *LC* leg curl, *LE* leg extension, *LP* leg press, *min* minutes, *RM* repetition maximum, *s* seconds, *SCR* seated calf raise, --*-* information was not reported by authors

### Meta-analysis

**Fig. 2:**
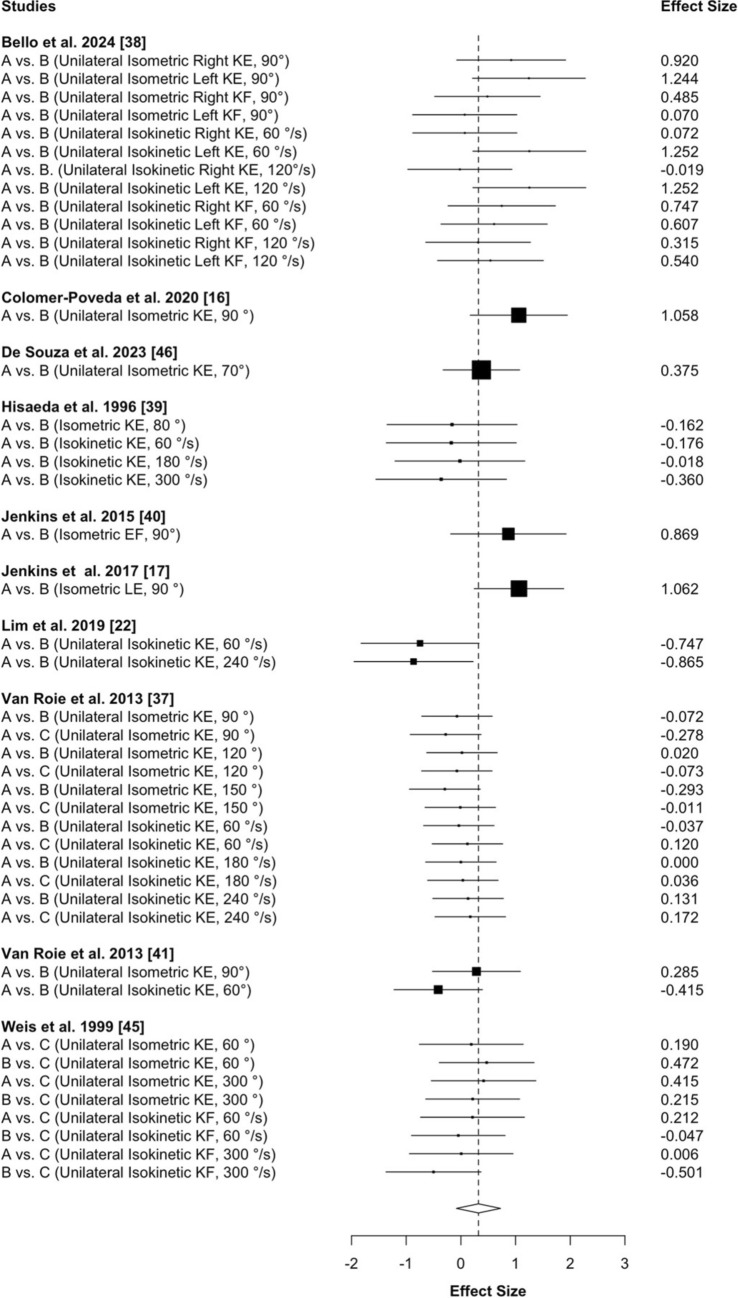
Forest plot depicting the effect size estimates for high- versus low-load isotonic resistance training on changes in non-specific strength. The model was estimated using effect size values calculated from the change score standard deviations (SDs). The black boxes symbolize the point estimates from each study. The horizontal lines symbolize the length of the 95% confidence intervals of the study result. The white diamond symbolizes the pooled estimates result, with greater changes for high-load training represented by a positive effect size, and greater changes for low-load training represented by a negative effect size. The overall effect is included at the bottom of the plot as a diamond with a width equivalent to the confidence interval for the estimated effect. *A* Training Protocol “A”, *B* Training Protocol “B”,* C* Training Protocol “C”, *KE* knee extension, *KF* knee flexion, *EF* elbow flexion, *LE* leg extension

The model using ES values calculated from the change score SDs resulted in 44 ES from ten studies (245 total participants; high load, *n* = 114; low load, *n* = 131). The overall ES (Cohen’s d) was 0.322 with a standard error of 0.17, and a 95% confidence interval of − 0.08 to 0.72 (Fig. 2, *p* = 0.104). The I^2^ was 44 and the Tau^2^ was 0.15. A sensitivity analysis demonstrated that the effect was stable across different values of Rho. The 95% prediction intervals ranged from − 0.45 to 1.1. The analysis using pre-SDs resulted in similar conclusions (Fig. S1 of the ESM).

### Risk of Bias Assessment

**Fig. 3:**
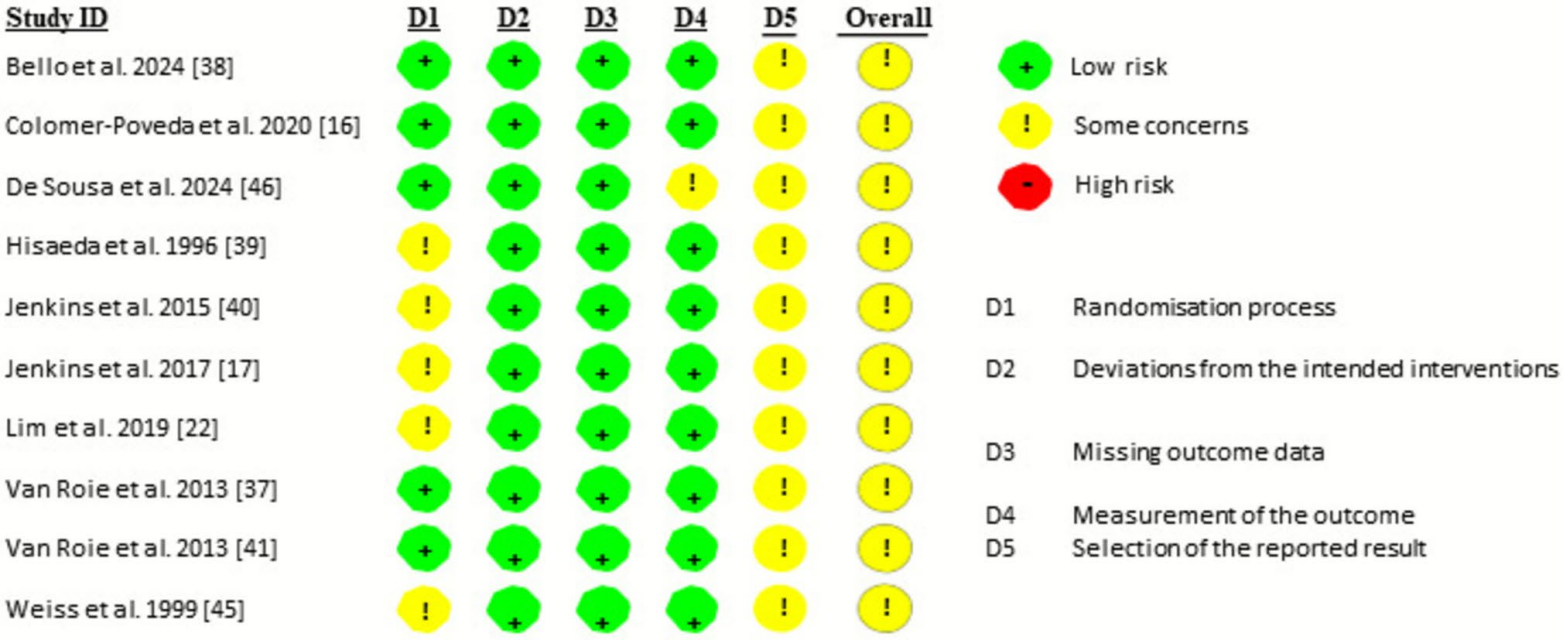
Individual risk of bias of the studies included in the present analysis, as evaluated according to the second version of the Cochrane risk-of-bias tool for randomized trials (RoB 2)

All ten of the studies included in the present meta-analysis were classified as having some concern for risk of bias, namely because of the randomization process (i.e., many did not report concealment allocation), and lack of pre-registration (Fig. 3).

## Discussion

The effectiveness of high-load resistance training for increasing task-specific strength has been extensively documented [5, 11, 13, 35], and is likely explained by adaptations underlying the principle of training specificity. In comparisons between high- and low-load resistance training, it has thus become common for researchers to include non-specific strength tests, presumably in an attempt to minimize the influence of training specificity [36] and better understand how different loading strategies impact strength development [4, 16, 18, 19, 22]. A previous meta-analysis by Schoenfeld et al. [12] reported no statistically significant differences between high- and low-load isotonic resistance training on changes in isometric strength. Those authors [12] were unable to quantify changes in isokinetic strength, and pointed out that a relatively low number of studies included measurements of isometric strength, which may have limited the ability to draw firm conclusions on the topic. In the current project, we pooled and analyzed 44 total ES values (i.e., 20 isometric and 24 isokinetic) from ten studies, which is over 20 more than the aforementioned meta-analysis [12]. Despite a lack of statistical evidence to suggest that high- and low-load isotonic training resulted in statistically significant differential changes in non-specific strength (*p* = 0.104), the overall ES value was 0.322, with a 95% CI between − 0.08 and 0.72. It is thus difficult to confidently conclude whether or not high-load training had greater effects than low-load training; however, the effect was biased in that direction. The prediction intervals ranged from − 0.45 to 1.1, which further highlights the inconclusiveness of the current literature.

While part of the heterogeneity in the response between high- and low-load training may be ascribed to methodological differences across the highlighted literature, the number of studies included in the current analysis limited our ability to explore any such moderating effects. Of note, seven of the ten studies [17, 22, 37–41] included in the present analysis employed multiple task-specific strength tests throughout their respective training interventions, and three of those studies [17, 38, 40] also assessed non-specific strength at pre-, mid-, and post-intervention. Given that frequent strength testing appears to induce a “training” stimulus capable of influencing task-specific strength gains [7, 42], it is entirely plausible that such methodologies may have attenuated the overall effect of high- versus low-load isotonic training on changes in non-specific strength. Of course, it may also be that repeated task-specific strength testing has only marginal effects on the transferability of strength adaptations, and that high-load isotonic training to task failure is not inherently superior to low-load isotonic training to task failure for increasing non-specific strength. In our opinion, however, the current state of literature does not allow for a definitive conclusion on the topic. After all, any additional exposure to maximal strength testing during a low-load training intervention would arguably change the training intervention from solely low-load training to a combination of both high- and low-load training [7].

Apart from any effect that frequent strength testing may (or may not) have had on changes in non-specific strength, it is possible that the results of the present analysis can be explained by an increased difficulty in detecting strength increases as the task becomes further removed from the actual training [8, 43]. Indeed, a recent meta-analysis [8] found evidence of a “generality of strength adaptation,” whereby isotonic resistance training was sufficient to increase isokinetic and isometric strength (i.e., non-specific strength) compared with a non-exercise control group. The effect, however, was much smaller than that observed for task-specific strength, which led the authors [8] to conclude that it may be difficult for a single study to meaningfully investigate the change in non-specific strength. To illustrate, based on the Spitz et al. [8] meta-analysis, using the lower bound estimate of 0.22 would require 326 participants per group to detect a change in non-specific strength compared with a time-matched non-exercise control group, whereas a change in task-specific strength would require just 12 participants per group. It is likely that the difference in effect between high- and low-load isotonic resistance training would be even smaller and thus, even more difficult to detect than if compared with a time-matched non-exercise control group.

The current project is not without limitations, perhaps the most notable being that none of the included studies was designed with the primary aim to investigate changes in non-specific strength (i.e., the current project is an analysis of secondary and/or tertiary outcomes). Another potential limitation is that nine of the ten included studies recruited non-resistance-trained participants focused on lower body testing outcomes, which restricts the generalizability of the findings to trained populations and upper body upper resistance exercise. In addition, we did not make the changes in non-specific strength relative to a time-matched non-exercise control group, which limits the ability to know whether the effects of high- and low-load isotonic training exceeded that of measurement error (i.e., systematic bias, random biological variability) [44]. On that note, however, it should be acknowledged that Weiss et al. [45], Colomer-Poveda et al. [16], and De Sousa et al. [46] were the only studies to include a time-matched non-exercise control group. Moreover, as discussed above, many of the studies [17, 22, 37–41] included herein employed multiple task-specific strength tests throughout the training intervention, and a few of them [17, 38, 40] also assessed non-specific strength at pre-, mid-, and post-intervention. A final limitation relates to our literature search. We elected to search only three databases and deployed a search strategy that was molded from previous reviews and meta-analyses [7, 12, 23]. It is conceivable that using more than three databases would have resulted in more studies being included in the analysis. However, given that our search strategy yielded nearly 7000 unique articles (i.e., ~ 4800 in the initial search and an additional 2100 in the updated search), which were all screened for analysis, we believe that our search captured a substantial portion, if not the entirety, of the available literature on high- versus low-load isotonic resistance training. Indeed, it has been shown that the benefit of adding more databases diminishes significantly after the first few major databases are searched (i.e., the number of additional unique relevant studies identified beyond a core set of databases was minimal) [47].

## Conclusions

The results of the current analysis were inconclusive as to whether high- and low-load isotonic training induced differential changes in non-specific strength, as demonstrated by the poor precision of the estimate and width of the prediction intervals. It should be acknowledged, however, that the overall ES appeared to be biased toward favoring high-load isotonic training, and that the majority of the studies included herein employed multiple strength tests throughout the training period. We interpret these data as tentative evidence suggesting that high-load isotonic resistance training may be more effective than low-load isotonic training for promoting strength adaptations in unpracticed movements (i.e., non-specific strength). Given that the previous literature has consistently shown greater increases in isotonic strength following high- versus low-load isotonic resistance training [7, 12, 13], a practical application of these findings is that individuals seeking to increase overall strength will likely benefit most from performing high-load isotonic resistance training. That said, additional research implementing a time-matched non-exercise control group and a direct comparison between low- and high-low isotonic resistance training without retesting maximal strength remains warranted. As more data become available, future work might also consider exploring the effects of potential moderating variables on changes in non-specific strength following high- and low-load isotonic training such as whether varying percentage ranges of maximal strength, training to task failure, and/or the type of non-specific strength testing (i.e., isokinetic or isometric strength test) influences outcomes.

## Supplementary Information

Below is the link to the electronic supplementary material.

Supplementary file1 (PDF 433 KB)

Supplementary file2 (PDF 127 KB)

## Declarations

### Availability of Data and Material

Data are available upon request from the corresponding author.

### Code Availability

The code is available upon request from the corresponding author.
