# Supplementary material for: Non-Specific Strength Changes Between High- and Low-Load Isotonic Resistance Training: A Systematic Review and Meta-Analysis
Source: Sports Med. 2025 Dec 12;56(3):763–73. doi: 10.1007/s40279-025-02370-8 (PMC13018011; doi:10.1007/s40279-025-02370-8)

Supplementary Figure S1. Forest plot depicting the effect size estimates for high- versus low-load isotonic resistance training on changes in non-specific strength. The model was estimated using ES values calculated from the Pre SD's. The black boxes symbolize the point estimates from each study. The horizontal lines symbolize the length of the 95% confidence intervals of the study result. The white diamond symbolizes the pooled estimates result. *A* Training Protocol "A", *B* Training Protocol "B", *C* Training Protocol "C", *KE* Knee Extension, *KF* Knee Flexion, *EF* Elbow Flexion, *LE* Leg Extension. The model using ES values calculated from the Pre SD's resulted in 24 ES from 8 studies (200 total participants; high-load, n = 89; low-load, n = 111). The numbers differ between models because Weiss et al. [45] nor Bello et al. [38] reported the Pre SDs in their manuscript. We reached out to authors from both manuscripts requesting their data. However, Weiss et al. [45] did not respond to our email requests for their data, and Bello et al. [38] were only willing to send the change scores and SD of the change scores. The overall ES (Cohen's d) was 0.159 with a standard error of 0.102, and a 95% confidence interval of -0.08 to 0.407 (p=0.169). The I^2 and Tau^2 were both 0. Sensitivity analysis demonstrated that the effect was stable across different values of Rho.

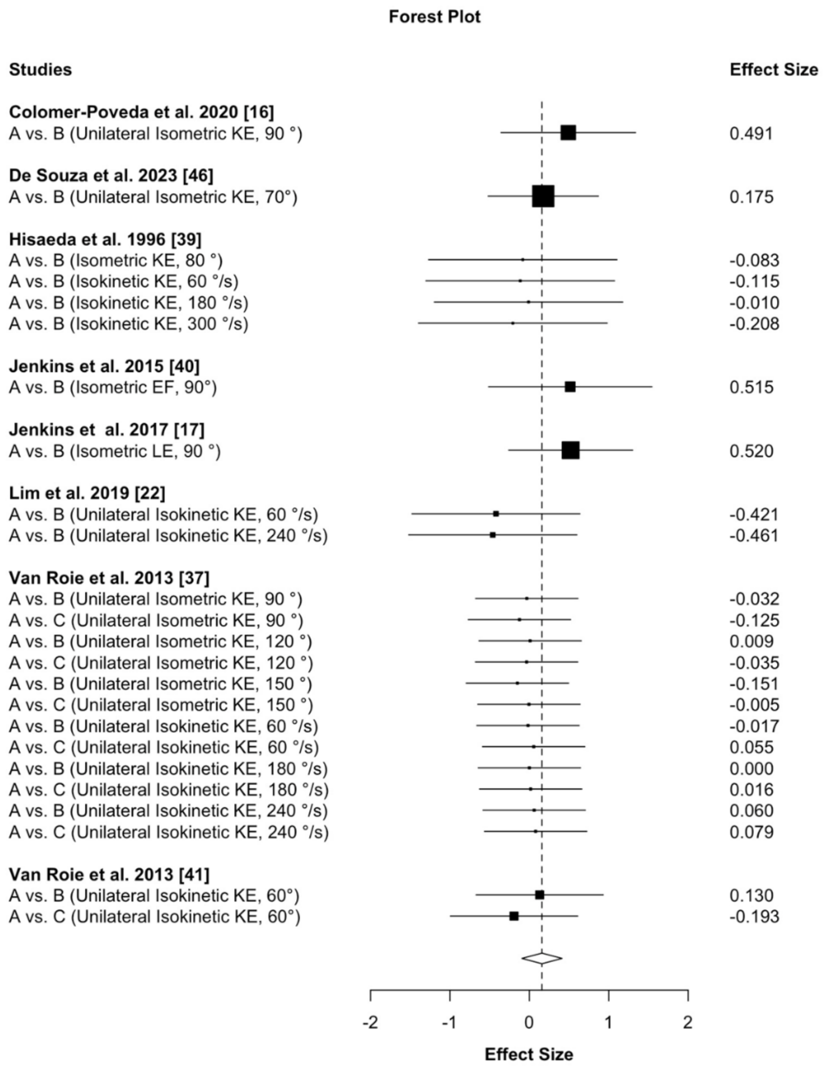

Supplement: Supplementary file 1 — Supplementary file1 (PDF 433 KB) [file 40279_2025_2370_MOESM1_ESM.pdf]
