# Supplementary material for: Non-Specific Strength Changes Between High- and Low-Load Isotonic Resistance Training: A Systematic Review and Meta-Analysis
Source: Sports Med. 2025 Dec 12;56(3):763–73. doi: 10.1007/s40279-025-02370-8 (PMC13018011; doi:10.1007/s40279-025-02370-8)
Supplement: Supplementary file 2 — Supplementary file2 (PDF 127 KB) [file 40279_2025_2370_MOESM2_ESM.pdf]

Supplementary Table S1. Papers that were excluded and the reasons why they were excluded.

Reference	Title	Reason
Akyüz et al. 2021	The effects of various strength training intensities on blood cardiovascular risk markers in healthy men	Did not include a non-specific strength test (or report data).
Grosset et al. 2014	Influence of exercise intensity on training-induced tendon mechanical properties changes in older individuals	Did not include a non-specific strength test (or report data).
Thuwakum et al. 2017	Low-load resistance training with hypoxia mimics traditional strength training in team sport athletes	Low load training groups exercised in hypoxia.
Popov et al. 2006	Hormonal adaptation determines the increase in muscle mass and strength during low-intensity strength training without relaxation	Low load training group exercised in smaller range of motion.
Netreba et al. 2007	Physiological effects of using the low intensity strength training without relaxation in single-joint and multi-joint movements	Not published in English Language.
Marshall-McKenna et al. 2021	Resistance exercise training at different loads in frail and healthy older adults: A randomised feasibility trial	Tested isometric strength on the same equipment (i.e., a "myometer").
Jones et al. 2021	The Effects of Varying Resistance-Training Loads on Intermediate– and High–Velocity-Specific Adaptations	Did not include/report a non-specific strength test (jumping).
Netreba et al. 2009	Physiological Effects of Low-Intensity Strength Training without Relaxation	Low load training group exercised in smaller range of motion.
Heinonen et al. 1993	Effects of equivolume strength training programmes of low, medium and high resistance on maximal iosmetric strength in sedentary women	Participants did exercises primarily on their own (i.e., non-supervised).
Ikezoe et al. 2020	Effects of Low-Load, Higher-Repetition vs. High-Load, Lower-Repetition Resistance Training Not Performed to Failure on Muscle Strength, Mass, and Echo Intensity in Healthy Young Men: A Time-Course Study	Exercise trained and tested on the same device (i.e., isotonic training and isometric testing on Biodex dynamometer)

Van Roie et al. 2017	Training load does not affect detraining's effect on muscle volume, muscle strength and functional capacity among older adults	Data were already been reported in another publication.
Rodriuguez-Lopez et al. 2021	Neuromuscular adaptations after 12 weeks of light- vs. heavy-load power-oriented resistance training in older adults	Within-subject design.
Fink et al. 2016	Impact of high versus low fixed loads and non-linear training loads on muscle hypertrophy, strength and force development	Participants did not refrain from their usual gymnastics training.
Njemini et al. 2017	Sex difference in the heat shock response to high external load resistance training in older humans	Data were already been reported in another publication.
Aagaard et al. 1996	Specificity of training velocity and training load on gains in isokinetic knee joint strength	High and low load training groups exercise on a hydraulic machine (i.e., involving concentric muscle contractions only).
Letieri et al. 2018	Effect of 16 weeks of resistance exercise and detraining comparing two T methods of blood flow restriction in muscle strength of healthy older women: A randomized controlled trial	Unclear as to the exercise prescribed for low load training protocol.
Leopoldine Onambélé-Pearson et al. 2010	Influence of exercise intensity in older persons with unchanged habitual nutritional intake: skeletal muscle and endocrine adaptations	Supervised and non-supervised training (i.e., one supervised gym-based class and two home-based sessions per week in low load training group versus two supervised gym- based classes and one home-based session per week in the high load training group).
Mertz et al. 2021	The effect of daily protein supplementation, with or without resistance training for 1 year, on muscle size, strength, and function in healthy older adults: A randomized controlled trial	Low-load training groups performed light-load home-based resistance 3–5 times weekly, using rubber bands, as well as their body weight for exercises chosen to mimic the muscle groups and movements used in training those assigned to the higher load training groups.

Pelet and Orsatti 2021	Effects of resistance training at different intensities of load on cross-education of muscle strength	High load training group also performed low load training.
Beneka et al. 2005	Resistance training effects on muscular strength of elderly are related to intensity and gender	Reported data by male and females.
Bemben et al. 2000	Musculoskeletal responses to high- and low-intensity resistance training in early postmenopausal women	Did not include a non-specific strength test (or report data).
Ogasawara et al. 2013	Low-Load Bench Press Training to Fatigue Results in Muscle Hypertrophy Similar to High-Load Bench Press Training	Within-subject design.
Kalapotharakos et al. 2004	The Effects of High- and Moderate-Resistance Training on Muscle Function in the Elderly	Did not include a low-load protocol (i.e., <60% 1RM).
Tanimoto and Ishii 2006	Effects of low-intensity resistance exercise with slow movement and tonic force generation on muscular function in young men	Authors did not present raw data, nor respond to email.
Lasawati et al. 2015	Low-intensity exercise with blood flow restriction increases muscle strength without altering hsCRP and fibrinogen levels in healthy subjects	Authors did not prescribe both exercise protocols to task failure.
Hortobágyi et al. 2001	Low-or high-intensity strength training partially restores impaired quadriceps force accuracy and steadiness in aged adults	Authors did not prescribe both exercise protocols to task failure.
Fink et al. 2018	Effects of rest intervals and training loads on metabolic stress and muscle hypertrophy	Did not answer email request.
Review Reopened		
Øfsteng et al. 2024	Superiority of High-Load vs. Low-Load Resistance Training in Military Cadets	Performed training/aerobic exercise alongside resistance training intervention.
Nakatani et al. 2024	Resistance training leading to repetition failure increases muscle strength and size, but not power-generation capacity in judo athletes	Performed judo training alongside resistance training intervention.
Fraga-Germade et al. 2024	Effect of Resistance Training Programs With Equated Power on Older Adults' Functionality and Strength: A Randomized Controlled Trial	Authors did not prescribe both exercise protocols to task failure.
